# Multisensory GPS impact on spatial representation in an immersive virtual reality driving game

**DOI:** 10.1038/s41598-022-11124-9

**Published:** 2022-05-05

**Authors:** Laura Seminati, Jacob Hadnett-Hunter, Richard Joiner, Karin Petrini

**Affiliations:** 1 Department of Psychology, University of Bath, Claverton Down, Bath, BA2 7AY UK; 2 Department of Computer Science, University of Bath, Claverton Down, Bath, BA2 7AY UK; 3 CAMERA and REVEAL Research Centers, Bath, UK

**Keywords:** Psychology, Risk factors

## Abstract

Individuals are increasingly relying on GPS devices to orient and find their way in their environment and research has pointed to a negative impact of navigational systems on spatial memory. We used immersive virtual reality to examine whether an audio–visual navigational aid can counteract the negative impact of visual only or auditory only GPS systems. We also examined the effect of spatial representation preferences and abilities when using different GPS systems. Thirty-four participants completed an IVR driving game including 4 GPS conditions (No GPS; audio GPS; visual GPS; audio–visual GPS). After driving one of the routes in one of the 4 GPS conditions, participants were asked to drive to a target landmark they had previously encountered. The audio–visual GPS condition returned more accurate performance than the visual and no GPS condition. General orientation ability predicted the distance to the target landmark for the visual and the audio–visual GPS conditions, while landmark preference predicted performance in the audio GPS condition. Finally, the variability in end distance to the target landmark was significantly reduced in the audio–visual GPS condition when compared to the visual and audio GPS conditions. These findings support theories of spatial cognition and inform the optimisation of GPS designs.

## Introduction

The sense of orientation and the ability of constructing a unified spatial mental representation, or cognitive map of the environment, originates from navigation^1,2^. Navigation includes the process of accurately defining a person’s own position in the environment and the activity of planning, taking and following a route. Hence, navigation in space is a skill that human beings have developed for survival and has evolved over the years^3^. Currently, however, this skill is required less because of the use of GPS devices. The use of GPS systems to navigate through space may have serious implications for an individual’s ability to create and develop rich mental spatial representations. Burnett and Lee^4^ underlined the risk of dependency on an external source of navigation information and of the importance of further investigation on the impact of GPS. Several studies have compared path learning using navigation assistance systems and mobile maps with paper maps or direct experience and have shown that paper maps provide better spatial knowledge and wayfinding performances^5–7^. Research comparing different types of spatial navigation technologies (mobile maps, augmented reality and voice) with no navigational aid have shown a poorer spatial knowledge acquisition when using spatial navigation technologies^8^. Finally, Ruginski et al.^9^ reported a negative association between long-term GPS use and mental rotation and perspective-taking abilities.

There are various reasons for these negative effects of GPS use^5,10–13^. First, GPS-based navigation is passive and thus does not require mental effort and control over action, impairing wayfinding performances (a cognitive component of navigation)^13,14^. GPS use might also disengage people from their surrounding environment, which reduces users learning^14^. Second, assisted navigation affects attentional mechanisms by directing navigator’s attention to the GPS device, rather than the surrounding environment^12,15,16^. For example, Hejtmánek et al.^12^ established that poor spatial acquisition was linked to the effect of GPS on people’s attention and not to individual differences in navigation cognitive skills. Moreover, Hejtmánek et al.^12^ showed that the amount of time spent using a GPS device negatively affected participant’s evaluation of their own navigation skills. Hence, navigational aids divide attention which impairs user’s learning of the environment^10,11^.

However, so far studies examining the effect of GPS and navigation aids on spatial cognition abilities have mostly focused on the effects of unisensory navigational aids. For example, research has shown the negative impacts of auditory aids on spatial information acquisition^17^ and demonstrated, in a population of patients with mild Alzheimer, how visual GPS (a mini display on the bonnet showing a directional arrow) are less effective compared to audio instructions^18^. Finally, studies have shown that visual GPS’s has a greater negative impact on navigational performances and spatial acquisition compared to audio GPS^12,15,16^. Hence, examining multimodal GPS systems could assist in understanding whether receiving redundant spatial information from multiple channels may counteract the negative effects of single modality GPS on the creation of spatial representations, by reducing uncertainty^19–21^.

Research in the field has mostly relied on the use of driving simulators or desktop environments and used tasks such as virtual pointing or landmark recall and thus does not account for proprioception and self-motion. IVR, using, for example, head mounted displays (HMD), tends to avoid this issue by allowing users to navigate the environment with proprioceptive (e.g., perceiving acceleration) and self-motion information as in real life^21–25^. Thus, IVR has expanded the potential of experimental design in spatial navigation and cognition, creating ecological paradigms by allowing the testing of participants in unfamiliar and complex environments, and effectively simulating self-motion. The advantage of IVR compared to desktop VR has been shown by studies which assessed that spatial knowledge acquired in IVR is comparable to knowledge acquired in real environments and can be transferred to real environments^26,27^. However, a possible adverse effect of IVR is motion sickness or visually induced motion sickness (VIMS), also known as cybersickness. Symptoms of sickness in IVR can include nausea, eye strain and dizziness and the causes can be linked to users, devices and stimulation characteristics^28,29^.

Navigation simulation studies have also shown the effectiveness of multisensory feedback, however currently we do not know whether receiving navigational aids from more than one sensory modality is beneficial for users, because it reduces sensory uncertainty as proposed by multisensory integration theories (e.g., Alais and Burr^19^; Ernst and Banks^20^). Multisensory stimuli have been used in the design of driving alert signals and research has shown that visual cues can be limited and counterproductive compared to auditory and tactile information^30^. Auditory and tactile signals permit a more rapid response than visual stimuli^31^ and are effective in drawing the user’s attention^32^, without significant attentional decrement^33–35^. Other studies have shown that integrating different warning sensory cues was more effective than using the unimodal cues when avoiding collisions^36^, and that a multisensory navigation system utilising visual, auditory and tactile cues is more effective than a system with single visual or audio–visual cues^37^. In addition, it has been shown that navigation performance improves when both visual and auditory aids were presented together rather than in isolation^38^, and that higher level of efficacy can be achieved with multisensory feedback rather than with visual feedback when promoting eco-sustainable driving behaviour^39^. Park et al. ^37^ and Smyth^38^ focused on assessing performances during navigation, by observing how long participants took to respond to unexpected road events, and Pietra et al.^39^ assessed possible reductions in fuel consumption thanks to multisensory feedbacks. However, the effect of using a multisensory GPS system on the formation of a cognitive map and/or spatial navigation remains unclear.

Furthermore, it remains unclear how individual differences and preferences in spatial representation can affect the effective use of different GPS systems. In fact, the effects of different GPS systems on cognitive map formation and navigation abilities have been shown to depend on individual characteristics. For example, Baldwin^40^ showed how individuals with survey wayfinding preference (representing the environment based on element locations and their relationships) and those with good spatial abilities benefitted from visual-map navigational aids, but only if provided in isolation from audio information. In contrast, individuals with route wayfinding preference (representing the environment as sequences of actions and landmark locations) or with low spatial abilities benefitted more from audio than visual information. In addition, experience in playing videogames can improve abilities involved during navigation, such as maintaining divided attention, visual acuity, contrast sensitivity and eye-hand motor coordination^41^. Previous studies have found a significant correlation between videogame experience and performance with many virtual tasks^42,43^, and it has been shown how experience with games with navigation and orientation tasks develops more efficient navigational strategies, due to practice^41^. Hence, individual differences in videogame experience could determine which type of GPS system would be more effective. Finally, effects of age and gender have been reported in relation to spatial information acquisition, performance in navigation tasks and IVR experience^22,44–48^. Given the relation between these individual differences and spatial navigation abilities it is important to consider them when assessing the effectiveness of different GPS modalities.

Hence, the present study used IVR to examine whether the use of a multisensory GPS system would counteract the negative effects of using visual only or auditory only systems and to observe the relation between GPS type and individual preferences in spatial acquisition. Specifically, the impact of a multimodal GPS on participants’ spatial representation and wayfinding performance was compared to that of a single modality GPS and of no GPS by using an IVR driving game. No study to date has compared wayfinding performances with different sensory navigation aids with wayfinding performance without any navigation aids, the inclusion of this baseline condition in the present study allowed us to better understand the effects of the different GPS systems on spatial navigation and representation abilities.

Based on previous findings on multisensory feedbacks during navigation and effects of unimodal sensory navigational aids, we hypothesised that participants would show (1) higher accuracy and precision during navigation without a GPS system than with either a visual or auditory GPS. We also predicted that participants would show (2) higher accuracy and precision in the audio–visual GPS condition than either in the auditory or visual GPS condition. Finally, we predicted that (3) individual preferences for either survey or route wayfinding will predict the performance in visual and auditory GPS respectively, while the examination of the effects of other individual spatial abilities on the use of the different GPS systems remained exploratory.

## Methods

### Design

We used a within-subjects design with two factors: (i) GPS type (No GPS, audio GPS, visual GPS, and audio–visual GPS) and (ii) route (five different routes with different levels of complexity and length), to assess the effect of different types of GPS on spatial representation and consequent navigation performance. We also had a number of predictors (gender, age, virtual experience, general orientation, use of cardinal points, survey preference, landmark preference, route preference) to assess their contribution to participants navigation performance. The dependent variables were “end distance” error (distance in meters between the correct landmark location and the end location chosen by the participant), “variation in end distance” (how variable was the distance between the correct landmark and the location chosen by participants across the 5 different routes), “time” (time in seconds taken to get to the landmark), and “route deviation” (overall route deviation from the correct/learned encoding route).

### Participants

An a priori power analysis for an ANOVA repeated measures within-factors was carried out using G*Power 3.1^49^ to estimate the required sample size. For the estimation we used a Cohen’s F of 0.25 (for a medium effect size), a level of power of 0.80, 1 group, 4 measurements, an alpha level of 0.05, and the adjustment to “Effect size specification as in SPSS”. The minimum sample size returned 24 if sphericity was met (1 in G*power) and 29 for some deviation from sphericity (0.75 in G*power). For the Friedman’s non-parametric test the calculated sample size was of 34 (29 + 29 * 0.15 = 33.35 round up to 34). However, given the exploratory nature of the study and the possible dropouts, we decided to have a bigger sample than the one estimated by the power analysis. A total of 45 (20 females) participants were recruited for the study. Participants’ age varied between 21 and 45 years old (*M* = 28.7, *SD* = 5.80). They all had a driving license and at least 3 years driving experience. Ten out of 45 participants did not complete the study due to motion sickness and the data for one participant had to be excluded because of missing data.

Hence the data included in the analysis were from 34 participants (15 females), aged between 21 and 42 years old (*M* = 24.5, *SD* = 5.72). The participants were recruited through leaflets and press advertisements. They were mainly undergraduate students, postgraduate students, and staff from the University of Bath.

### Apparatus and materials

**Figure 1:**
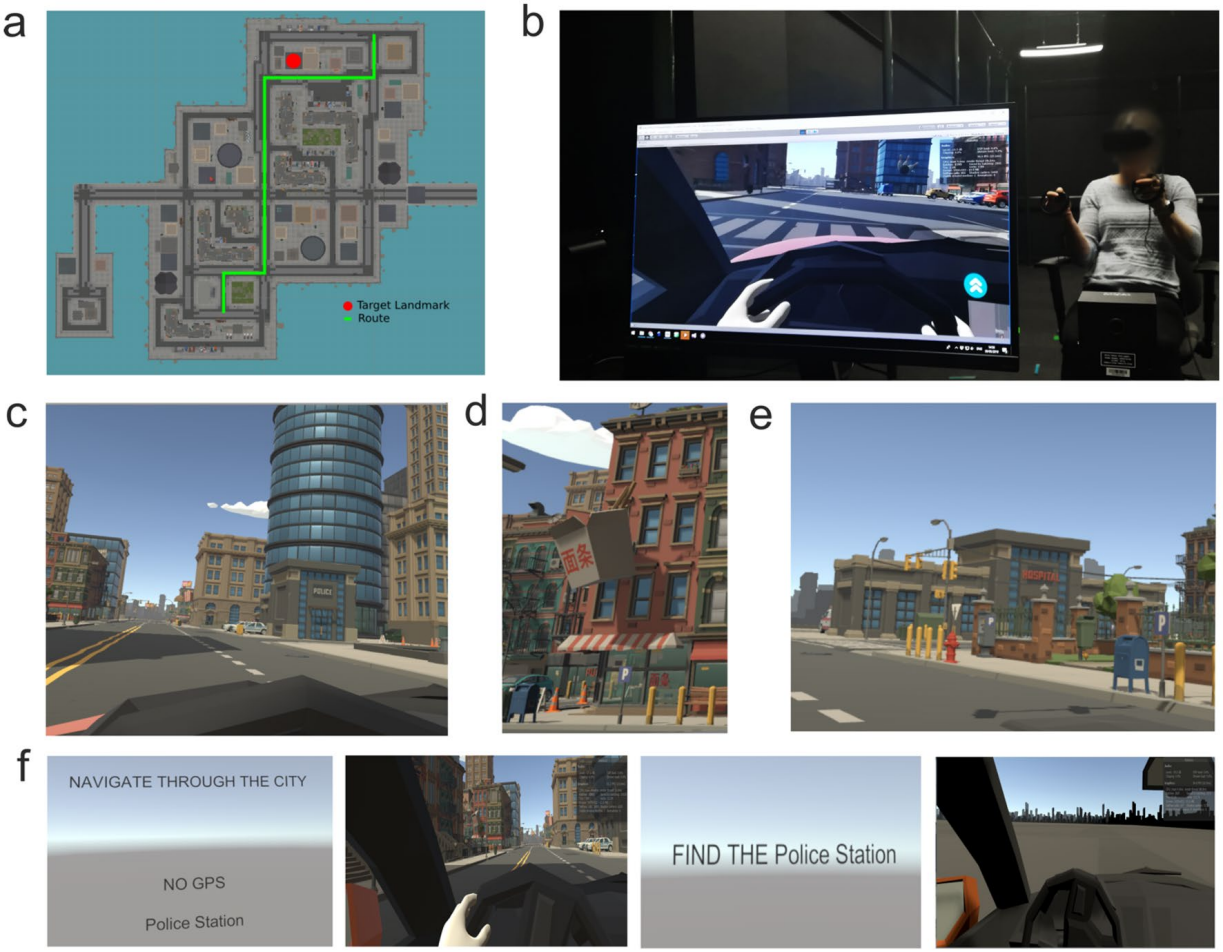
VR environment. (**a**) The top-down view of the IVR city environment with a route’s example (the route matches the trajectory in Fig. 2a). (**b**) The experimental setting. (**c**–**e**) Examples of target landmarks on the routes; “Police Station”, “Chinese Restaurant”, “Hospital” respectively. (**f**) Task representation and examples of participant’s view during the encoding and the test phase. In the example the participant was tested in the No GPS condition and was informed that the target he/she had to drive to at a later time was the police station (the route the participant drove through in this condition passed by the police station but did not stop there. During the test phase the participant was told by the screen to find the police station by driving in a blank city environment. To create Fig. 1. CorelDRAW 2020 (64-Bit) was used (https://www.coreldraw.com/en/pages/coreldraw-2020/).

The virtual city (Fig. 1a) used in the present study was presented in a Unity application using 3D models available from the Unity Asset Store. The PC hardware used for the experiment was an Alienware Desktop PC with GTX 1080 Ti graphics cards and the experimental setting can be seen in Fig. 1b. The 3D city included the five target landmark buildings (see Fig. 1c–e for examples) used for the test phase of the study (each one of the five targets was used for a single route). Width and length of the city were approximately 350 m and 400 m. As HMD we used the Oculus Rift CV1 (Oculus) that offered an immersive field of view of 90° horizontally. See Fig. S1 in the supplemental material for details about HMD and screen resolution. Oculus Touch controls were used to control the car and drive in the virtual city. Forward and reverse acceleration were mapped to the left controller joystick and were position-dependent (i.e., pushing the joystick further forward increased acceleration). Steering wheel rotation was mapped to the roll rotation of the right-hand controller. In this way, moving and rotating the right hand along the circumference of the virtual steering wheel produced an equivalent steering rotation with a 1:1 steering ratio.

**Figure 2:**
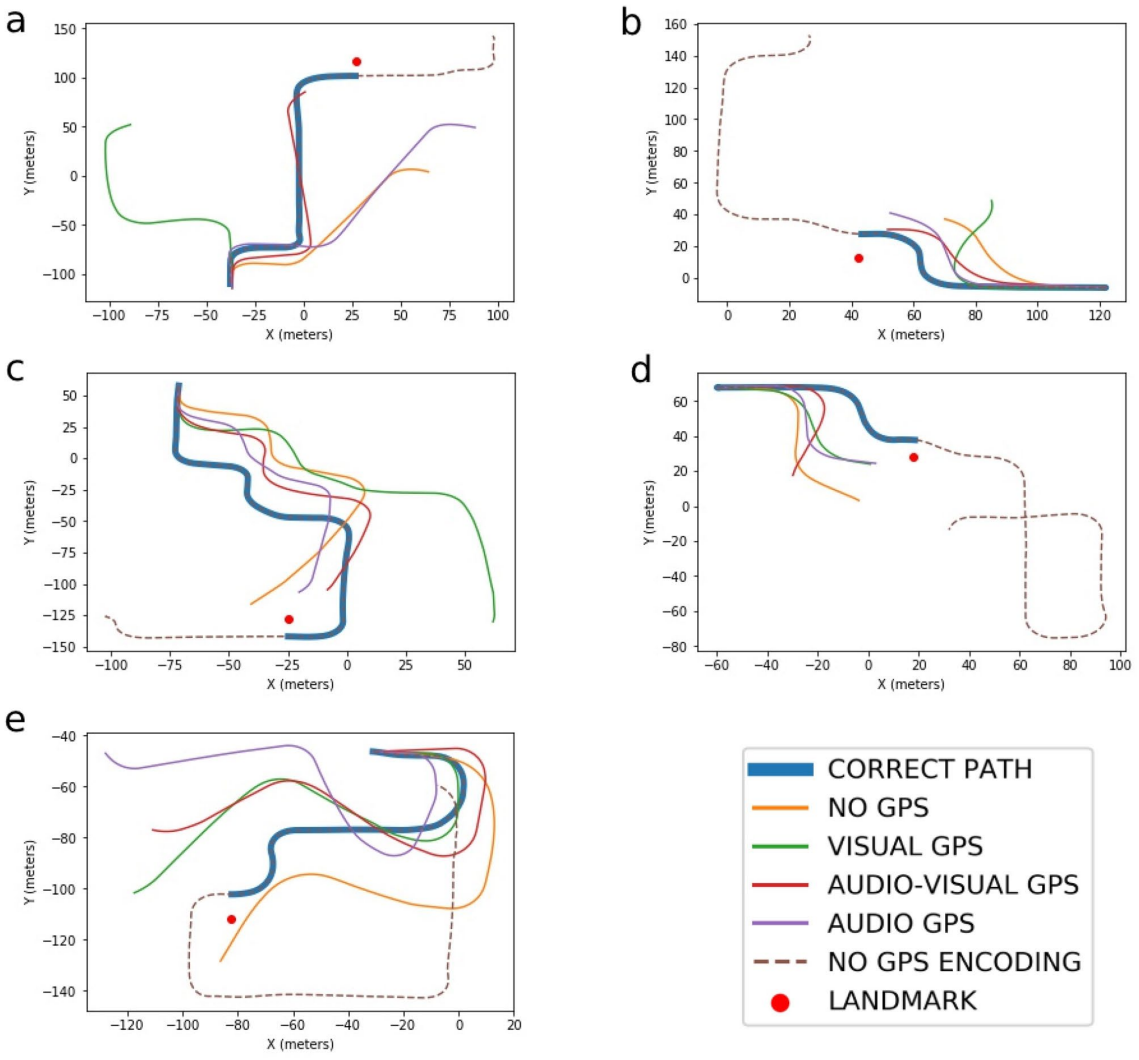
Examples of a participant’s performances in the test phase for the five routes (**a**–**e**) for each GPS condition. The dotted line refers to the entire route used in the encoding phase; the red point refers to the target landmark of the route. (**a**) matches the route shown in Fig. 1a.

The Questionnaire on Spatial Representation, developed by Pazzaglia, Cornoldi and De Beni^50^ was used to assess individual differences and preferences of spatial representation’s cognitive styles. It is an 11 item self-assessment questionnaire that measures different cognitive styles in spatial representation, general sense of direction, knowledge and use of cardinal points, and outdoor and indoor orientation ability organized in five factors. Factor 1 groups items on general sense of direction in open and closed environments (items 1, 2, 3c, 8, 9, 11), factor 2 represents the use of compass directions in orienting tasks (items 5, 6, 12), factor 3 represents preference for a survey representation of space (items 3c, 4a, 7a), factors 4 and 5 represent preference for landmark-centred (items 3b, 4c) and route spatial representation (items 3a, 4b), respectively (see Supplemental material). Scores are given on a Likert scale (from 1 = not at all to 5 = very good), with a reliability of 0.75 tested on a sample of 285 undergraduate students^50^. Pazzaglia and Taylor^51^ used this questionnaire in a study similar to the present study by investigating spatial acquisition in a virtual environment.

Videogame experience was measured using simple yes–no responses concerning participants’ ability to play videogames and their familiarity with them (see Supplemental material).

### Ethical approval

The research received approval from the Department of Psychology Research Ethics Committee of the University of Bath (Ethics approval’s reference number: 19-072). All research was performed in accordance with relevant guidelines and informed consent was obtained from all participants (see Supplemental materials).

### Consent to participate

All participants gave their consent to take part in the experiment by signing the information and consent forms (see supplemental materials).

### Consent for publication

All participants gave their consent to use the data collected from the study by signing the information and consent forms.

## Procedure

### Pilot tests

Two different pilot studies with 3 participants each, aged between 27 and 30 years old (*M* = 28.1, *SD* = 1.16) were carried out to optimise the design and the task used in the main study. Due to the pilot studies, we selected five routes out of ten to use in the main experiments thus greatly shortening the task and reducing fatigue for the participants. We selected the five routes that most differed among the ten used in the pilot, and the judgements on the level of difference among the routes was based on differences in length, number of turns and landmarks, and on the criteria that they should not have had any overlap in trajectory (i.e., they should cover different parts of the city). The pilot studies were also used to caliberate the delivery time for the GPS instructions in the main study, to make sure participants could use the GPS information effectively during the encoding phase and minimise participant confusion. Please see Supplemental material for a full description of the pilot studies.

### Experiment

Participants were welcomed and accompanied to the Virtual Reality Lab where the task was explained and participants gave their consent to participate. Participants were asked a few questions about age, gender, videogame experience (i.e., participants were asked to answer yes or no based on their ability to play videogames and the level of familiarity with them) and to fill out the Questionnaire of Spatial representation^50^. The task was once again verbally explained to the participants and they were shown how to use the two Oculus Touch controllers to drive the car in the IVR city. Participants were finally reminded that they had the possibility of taking breaks, or withdraw from the study, informing the experimenter in case of fatigue or motion sickness. Participants could take breaks and for as long as needed, pausing the experiment between the trials. During the pause, participants could take the HMD off, drink water and/or relax. Participants wore the Oculus Rift and started the task sitting on a chair (see Fig. 1b). The whole experiment lasted 45–50 min (approximately 10–15 min for completing the demographic information and the questionnaire and 30 min for completing navigational task). At the beginning of the task, the participants faced the virtual city environment from the virtual driver seat of a car.

The five different routes were randomly presented to each participant in each GPS condition (i.e., participants completed 20 trials overall: 4 GPS conditions × 5 routes/landmarks) and they had different lengths and difficulties. Some routes were longer than others, involving more complex navigations such as more turns, and containing more landmarks along them than others to maintain a high level of realism similarly to real-life city driving navigation. Each of the five routes started from a different start point in the city and the software selected them in a randomized order. In the audio, visual and audio–visual GPS conditions participants had to complete the routes following the GPS instructions and passing in front to a target landmark for each route. In the visual GPS condition participants could see a screen with a street map on the right of the steering wheel, that showed the car moving position on a 2D map (see Video 1 in the Supplemental material). They were requested to follow the arrow above the screen, which showed the direction of the route similarly to available GPS. As it can be seen in the video the GPS instructions slightly differed from those typically provided by a real GPS system, which usually delivers information about how far a turn is, and visually represents the entire path. However, with the city and road lengths being quite small, the relative distance between instruction and the required turn was not so small. Further, the driving speeds in the city were low, so making a turn soon after a GPS instruction was not difficult (participants had no issue in turning on time according to the pilot testing and observation during the experiment). In the audio GPS condition participants were informed of the route via audio cues. Participants were told to either “turn left”, “turn right”, or go “straight ahead” as they navigated the route. The audio cues were generated from an online text-to-speech resource with a British male voice profile. The visual GPS was not visible in this condition. In the audio–visual GPS condition both the visual GPS and the voice describing the route were available. Finally, in the No GPS condition participants did not drive and were sitting on the driving seat without control on the Oculus Touch controllers as if the car was driving on autopilot. No GPS was available to drive along the route as the participant was guided. This condition was chosen and developed based on the existing studies that used a driving VR experience to assess memory (e.g., Plancher et al.^52^). The No GPS condition was designed to simulate a self-driving car, where the participants actively experienced and see the route from a first-person perspective as in the other conditions but without the aid of a GPS system.

Each trial of the experiment comprised of an encoding phase and a subsequent test phase for every GPS condition and route. In the encoding phase, participants were driving through the 3D city following one out of five possible routes for one out of four possible GPS conditions in randomised order. Along each route of the encoding phase at least one target landmark was presented, and the participant was informed before starting the encoding phase which target landmark they would have to drive to at a later time during the test phase. Each encoding phase ended automatically; the endpoint of the five routes was pre-established in order to differentiate them for length, number of turns and number of landmarks. A screen before every encoding phase showed the name of the target landmark and the GPS condition (e.g., “No GPS / Police Station”, Fig. 1f).

In the test-phase, participants were placed again at the start point of the route and were asked to drive to the target landmark presented previously during the encoding route. Participants could not see the landmark they had to reach as they had to find their way based only on their formed spatial representation (i.e., through the formation of a cognitive map during the encoding phase; Petrini et al.^21^; Tcheang et al.^24^). For this reason, the IVR city was hidden and only the distant skyline was visible (Fig. 1f) in line with previous studies testing spatial knowledge acquisition and formation of cognitive maps in IVR by testing participants navigation performance in darkness^21,24^. If participants relied only on proprioceptive information when reforming the test phase and driving to the target landmark (i.e., if they considered only turns and distance travelled) then no difference in performance between the encoding GPS conditions would be found, as the five routes in each GPS condition contained the same number and type of turns and distances. When satisfied with the final location, that was supposed to be the location of the target landmark, participants had to press a button on the Oculus Touch. A screen before every test phase asked the participants to drive to the landmark, encountered during the encoding phase (e.g., “Find the Police Station”, Fig. 1f).

There was a practice phase before the real experiment to familiarise with the task, the environment and the controllers. During this practice phase all participants completed the trials (encoding phase and test phase) of the same route for all four GPS conditions and learned to effectively use the controllers. The practice was carried out in the same virtual city but using a different route from those used in the main experiment.

### Data analysis

Analyses were conducted in SPSS, “Statistical Package for Social Science”, (IBM SPSS Statistics 25). First, to decide which analysis was more appropriate we checked whether the data met or not the assumption of normal distribution and sphericity (see Supplement materials for details). The assumptions for a repeated measures ANOVA were not met for all these measures. Thus, to examine how different types of GPS affected participants’ navigation performances and spatial acquisition (hypothesis 1 and 2) we:

- collapsed the data across the five routes for “end distance” error, “time” and “route deviation” and carried out Friedman’s tests for all these measures with GPS conditions (No GPS, audio GPS, visual GPS, audio–visual GPS) as within-subjects factor. For the variability in end distance error, we carried out a one-sample Wilcoxon test to compare the median of each GPS condition to the median of the No GPS condition and a paired-samples Wilcoxon’s test to compare the variability in the audio–visual GPS condition to that in the audio GPS and visual GPS condition.

And to examine the contribution of different individual differences and spatial representation preferences on participants’ performance under different GPS conditions (hypothesis 3 and exploratory analysis) we:

- carried out a series of multiple linear regression analyses on the measures (end distance error, time and route deviation) that returned a significant effect of GPS in the previous analyses, with gender, age, videogame experience, general orientation, use of cardinal points, survey preference, landmark preference, route preference as predictors.

Measures of “end distance” error (distance in meters between the correct landmark location and the end location chosen by the participant), “time” (time in seconds spent to get to the landmark), “route deviation” (overall route deviation from the encoding route) were obtained from the test-phase to determine the level of accuracy in retracing the target landmark (see Fig. 2 for the representations of a participant’s performances). We utilized Dynamic Time Warping (DTW) as a measure of route deviation. DTW provides a measure of similarity between two temporal sequences. In this case, DTW provides a measure of similarity between the route participants encoded (were exposed to) during the learning phase of the experiment and the route they drove during the test phase, even if participants drove the route at different speeds and accelerations. DTW similarity scores were calculated using the FastDTW algorithm with Euclidean distance function^53,54^ in Python.

The variability (standard deviation) in “end distance” was also calculated for the different sensory conditions (audio, visual and audio–visual) to examine whether having more than one type of GPS in the audio–visual condition reduced uncertainty when compared to audio only and visual only conditions^19,20^. Finally, we examined for any learning effects developed by participants during the experiment.

## Results

### Distance to landmark location

**Figure 3:**
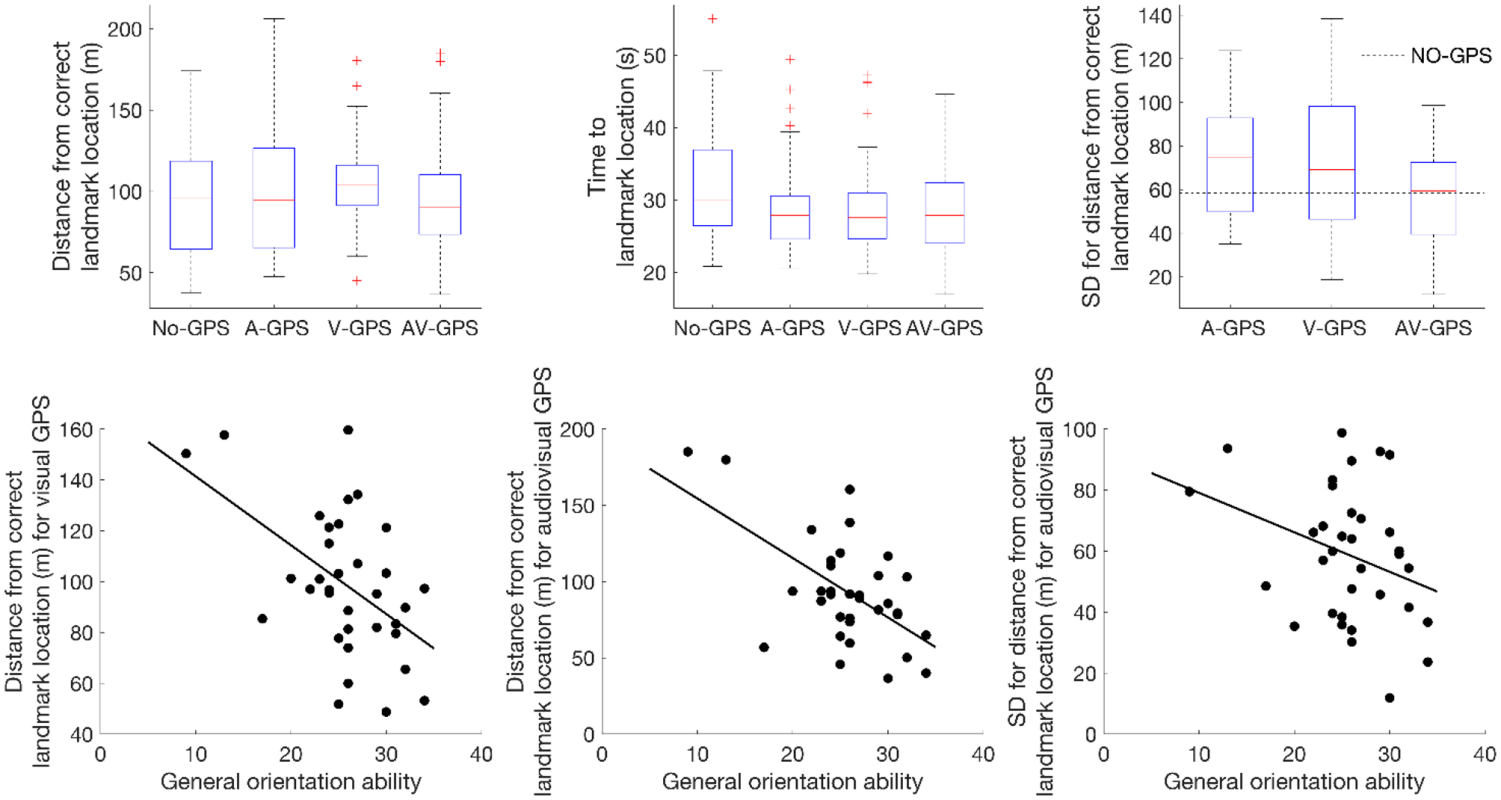
Results of the performances and general orientation ability in the different conditions. The left top panel shows a boxplot for end distance error for the different GPS conditions. The central top panel shows a boxplot for the time participant took to arrive to the target landmark for the different GPS conditions. The right top panel shows a boxplot for the standard deviation for the end distance error in the different GPS conditions. The bottom left panel shows the relation between individual “general orientation ability” and “end distance” error in the visual GPS condition, the bottom middle panel shows the relation between individual “general orientation ability” and “end distance” error in the audio–visual GPS condition, and the bottom right panel shows the relation between individual “general orientation ability” and “variability” in end distance error (or standard deviation) in the audio–visual GPS condition. The red line in the boxplot represents the median and the box the interquartile range (IQR). Note: Friedman’s and Wilcoxon’s tests are carried out by comparing mean ranks between conditions, but we reported medians and IQRs as descriptive measures as they are more commonly reported for non-parametric tests. See supplemental material for measures of mean rank.

The results of the Friedman’s test for “end distance” error (the distance between the target location and where participants stopped the car) returned a significant effect of “GPS condition”, ${\chi }^{2}$(3) = 10.80, *p* = 0.013, Kendall's *W* = 0.106. Multiple comparisons between GPS conditions, Bonferroni corrected, returned a significant difference between no GPS condition (*Mdn* = 96.17, *IQR* = 54.64) and visual GPS condition (*Mdn* = 104.29, *IQR* = 27.26), *p* = 0.029 and between audio–visual (*Mdn* = 89.98; *IQR* = 39.77) and visual GPS condition *p* = 0.029 (Fig. 3, left panel). A series of multiple linear regression analyses were then run for all the predictors of interest (gender, age, virtual experience, general orientation, use of cardinal points, survey preference, landmark preference, route preference) with end distance error in each GPS condition as outcome. Using the enter method the multiple linear regression analysis for no GPS condition returned a non-significant regression equation *F*(8, 25) = 1.39, *p* = 0.247, with an *R*^2^ of 0.303, while a significant regression equation was returned for audio GPS, *F*(8, 25) = 2.35, *p* = 0.049, with an *R*^2^ of 0.429, for visual GPS, *F*(8, 25) = 3.40, *p* = 0.009, with an *R*^2^ of 0.521, and for audio–visual GPS, *F*(8, 25) = 3.76, *p* = 0.005, with an *R*^2^ of 0.546. For the audio GPS condition only two of the predictors significantly predicted the end distance error, videogame experience (*β* = 0.50, *t* = 2.49, *p* = 0.020) and landmark preference (*β* = 0.38, *t* = 2.25, *p* = 0.034). For the visual GPS condition only one of the predictors significantly predicted the end distance error, Factor 1 of the Questionnaire on Spatial Representation^50^ on general sense of direction in open and closed environments that we labelled as “general orientation ability” (*β* = − 0.97, *t* = − 3.29, *p* = 0.003). Finally, for the audio–visual GPS condition only two predictors significantly predicted the end distance error, general orientation ability (*β* = − 0.84, *t* = − 2.92, *p* = 0.007) and route preference (*β* = 0.47, *t* = 2.49, *p* = 0.020).

### Time to landmark location

The results of the Friedman’s test for “time” (time taken by participants to arrive to the landmark location) returned a significant effect of “GPS condition”, ${\chi }^{2}$(3) = 14.82, *p* = 0.002. Multiple comparisons between GPS conditions, Bonferroni corrected, returned a significant difference between no GPS (*Mdn* = 30.04, *IQR* = 11.05) and audio GPS (*Mdn* = 27.88, *IQR* = 6.34) condition (*p* = 0.016) and between no GPS and audio–visual GPS (*Mdn* = 27.85, *IQR* = 8.56) condition (*p* = 0.002; Fig. 3, middle panel). A series of multiple linear regression analyses were then run for all the predictors of interest (gender, age, virtual experience, general orientation, use of cardinal points, survey preference, landmark preference, route preference) with time in each GPS condition as outcome. Using the enter method the multiple linear regression analyses returned non-significant regression equations for all GPS conditions, *F*(8, 25) ≤ 1.36, *p* ≥ 0.261, with an *R*^2^ ≤ 0.303.

### Route deviation from encoding route

The results of the Friedman’s test for route deviation from encoding route returned a non-significant effect of “GPS condition”, ${\chi }^{2}$(3) = 0.18, *p* = 0.981. Hence, no further analyses were performed.

### Variability in distance to landmark location

We tested whether the “end-distance error” across the five routes was less variable when given both visual and audio GPS information than only visual or only audio GPS information, as we would expect based on the reduction in variability usually achieved when combining information from more than one sense^19–21^. The results of the paired-samples Wicoxon’s test, Bonferroni corrected, returned a significant difference between audio (*Mdn* = 74.63, *IQR* = 43.57) and audio–visual (*Mdn* = 59.44, *IQR* = 35.05) GPS, *Z* = − 2.45, *p* = 0.028, *r* = 0.42, and between visual (*Mdn* = 69.14, *IQR* = 51.96) and audio–visual GPS, *Z* = − 2.49, *p* = 0.026, *r* = 0.43, i.e., having both audio and visual GPS did significantly reduce variability in performance (Fig. 3, top right panel). A one-sample Wilcoxon test, Bonferroni corrected, also showed that while visual GPS (*Z* = 2.16, *p* = 0.093) and audio–visual GPS (*Z* = 0.12, *p* = 0.905) did not significantly differ from the No GPS condition (*Mdn* = 58.40, *IQR* = 35.26), the audio GPS condition did significantly differ and its variation was higher than in the no GPS condition (*Z* = 2.50, *p* = 0.036, *r* = 0.43). A series of multiple linear regression analyses were then run for all the predictors of interest (gender, age, virtual experience, general orientation, use of cardinal points, survey preference, landmark preference, route preference) with variability in end distance error for each GPS condition as outcomes. Using the enter method the multiple linear regression analyses returned only one significant regression equation for the audio–visual GPS condition, *F*(8, 25) = 3.71, *p* = 0.005, with an *R*^2^ of 0.543, and similarly for the end distance error results only three predictors significantly predicted the variability in end distance error, general orientation ability (*β* = − 0.76, *t* = − 2.65, *p* = 0.014), landmark preference (*β* = 0.43, *t* = 2.81, *p* = 0.010), and route preference (*β* = 0.59, *t* = 3.11, *p* = 0.005). For all other GPS conditions no significant regression equation was found *F*(8, 25) ≤ 1.99, *p* ≥ 0.090, with an *R*^2^ ≤ 0.389.

### Learning effect

We examined whether there was any learning effect with increasing number of trials. We used regression analyses to examine whether “end-distance” error, “route deviation” and “time” decreased with practice. A linear regression was calculated to predict “end-distance” error based on the trial number and returned a significant regression equation *F*(1, 18) = 7.72, *p* = 0.012, with an *R*^2^ of 0.300 and *β* = − 0.55, thus showing an increase in accuracy in driving to the final target position with practice. Another linear regression was calculated to predict “time” based on the trial number and was significant *F*(1, 18) = 26.20, *p* < 0.001, with an *R*^2^ of 0.593 and *β* = − 0.77, thus showing that participants also took less time to drive to the target position with practice.

## Discussion

Using an IVR driving game, we tested a group of adult participants on a wayfinding task to investigate the influence of GPS navigation aids on spatial representation. We specifically asked whether combining a visual and audio GPS would overcome the negative effect of visual only and audio only GPS previously found in literature. In addition, we tested whether individual differences in spatial abilities and preferences would determine the efficacy of the different GPS navigation aids. Consistent with previous findings, we observed that visual only GPS reduced the level of accuracy when driving to the target landmark and negatively affected navigational performances and the formation of a spatial representation. Audio–visual GPS, as expected, increased accuracy (reduced error) when driving to the target landmark compared to visual only GPS but did not differ from auditory only GPS or without a GPS. Also, our results showed that in the audio–visual GPS condition the level of variability in performance was significantly reduced (i.e., precision in driving to the target increased) compared to both the visual only and auditory only GPS condition.

Our findings are consistent with those showing a benefit of multisensory driving alarms^36^ and multisensory aids during navigation in real time^37,38,40^. However, while Park et al. ^37^ and Smyth^38^ established the efficiency of audio–visual information during navigation, our study shows an improvement in cognitive spatial map creation when using audio–visual GPS rather than a visual only or auditory only GPS. Hence, using the two GPS guidance methods together in our study did counteract the negative effect of using either GPS in isolation (especially compared to the visual GPS). However, it is unclear why using a multisensory GPS cancels out the previously found negative effects of single modalities GPS. It could be because it reduces the passive use of these aids^13^, or it reduces people disengagement from their surrounding environment^14^, or the GPS effects on attentional mechanisms^12,15,16^, or as we hypothesised because it helps to create a richer and more precise cognitive map of the environment and routes by reducing sensory uncertainty and the possibly cognitive load. Future studies could examine whether one of these explanations or all of them are behind the benefits of multisensory GPS aids.

However, the benefit of the audio–visual GPS in our study did not exceed that of not using a GPS. This lack of difference between the multisensory GPS condition and the condition without GPS could be explained by differences in control over the car and decision-making between these two conditions when learning the city layout^55^. During the encoding phase of our study, participants had no control and no-decision making in the condition without GPS, whereas in the multisensory GPS condition participants had no decision-making but did have control. As control can be achieved by using local information rather than a spatial global representation^55^, the added control in the multisensory GPS condition could have negatively affected the formation of a spatial representation based on the driven route. This explanation is supported by a lack of difference between the two single modality GPS, which like the multisensory GPS condition had control but no decision making, and the condition without GPS. Hence, to examine whether the benefit provided by a multisensory GPS can exceed that of not using a GPS, a future study could have a condition with no GPS in which participants have control during the encoding phase rather than being passively guided. However, not having GPS aids significantly increased the time spent to reach the target landmark compared to when audio and audio–visual GPS instructions were available. Only visual GPS did not differ significantly from the condition with no GPS when considering the time taken to reach the target landmark. Hence, although an audio–visual GPS may not significantly improve the level of accuracy and precision in reaching a target landmark when compared to the condition with no GPS aid these results do suggest that an audio–visual GPS may significantly reduce the time to get to a chosen destination. Additionally, our condition without GPS is insightful because the use of autonomous driving vehicles is rapidly increasing, which potentially could have a negative impact on spatial acquisition^56^. Self-driving vehicles are likely to produce a degradation in spatial survey knowledge in people who drive frequently and do not usually ride as passengers^56^. However, as proposed by Qin and Karimi^56^, further controlled driving simulation’s experiments are needed to answer this question and to examine differences between supportive GPS devices and the use of autonomous driving vehicles in affecting spatial acquisition.

As expected, we also found some effects of individual differences on the formation of spatial representation and navigation performance. Individual “general orientation ability” increased with participants accuracy in driving to the target landmark, showing that participants with high level of general orientability were closer to the target landmark than participants with lower general orientation ability. This was especially evident for visual GPS and audio–visual GPS conditions. Furthermore, the distance to the target landmark for individuals that benefitted from the audio GPS was predicted by landmark-based orientation preference. These results support Baldwin’s^40^ theory that participants with good general orientation ability are better when using visual GPS than those with low general orientation ability, who generally prefer audio cues. Our results thus underline how visual GPS should be the least preferred option when the aim of the user and driver is to acquire a rich and useful mental representation or map of an unfamiliar environment (which will later allow the user to find their way with enhanced speed and accuracy in the same environment). Our results also suggest how GPS companies could include measures of orientation and spatial representation ability to their software settings to automatically determine a user profile and suggest the most beneficial GPS modality for that specific individual. Videogames experience significantly predicted the distance to the target landmark only in the audio GPS condition, suggesting that videogames experience helped processing auditory information^57^. This result differs from common findings that show mainly benefits in visual task performances for people with videogame experience (e.g., Stewart et al.^58^). Future studies could better distinguish between videogame experts and non-expert by increasing the number of questions about videogame experience instead of a single yes/no question. For example, questions about the age at which participants started to play videogames, how frequently they play and how many hours a week they play could be used, as well as questions about videogames characteristics (e.g., type of graphics and sound features) to better understand the relation between videogame experience and the ability to process different sensory cues when using navigational aids.

Finally, our prediction of a learning effect was supported by a decrease in time taken to locate the target landmark and an increase in accuracy in locating the target landmark with number of trials. Indeed, previous research found that environmental knowledge increases with repetition, regardless of methods used^13,59^.

## Limitations

Individuals using driving simulations often show symptoms of motion sickness (e.g., Yi et al.^18^), as do participants in IVR experiments^60,61^. In our study ten participants did not complete the experiment because of motion sickness, and this despite the six participants taking part to the pilot not reporting any motion sickness (even if the pilot was much longer than the main study). Based on precautions we normally use for IVR studies we included a practice phase before the experiment to habituate participants to the IVR environment and task. We also reduced the number of routes and target landmarks that we had previously planned to limit fatigue and we allowed participants to take breaks between the trials. Overall around 15 participants out of 34 took breaks—to drink water or just take the HMD off and relax for a few minutes—only four participants took more than one break. According to informal reports at the end of the experiment, no notable discomfort was reported by the thirty-four participants who finished the study. The majority of participants who did not complete the task because of motion sickness stopped quite early on during the study suggesting that these individuals were particularly sensitive to motion sickness. We can assume that high levels of motion sickness observed in our study occurred because of sensory-vestibular conflict^62^ as visual stimuli’s movement occurred without corresponding vestibular cues, given that participants were sitting on a chair while the car was moving. We know from previous studies that there are big individual differences in the amount of perceived discrepancy between self-motion and visual cues for the same IVR^21^, but according to informal reports from the participants, sensory-vestibular conflict generated dizziness, especially in the condition without GPS. However, we wanted to include the condition without GPS to have a baseline condition and to simulate autonomous driving vehicles, although an active no GPS condition could be tested in future studies to avoid the sensation of dizziness caused by the car’s autonomous movement. Motion sickness could also be related to low-resolution, hence future studies could use HMDs with higher resolution and overcome sensory-vestibular conflict using a driving motion simulator. Additionally a pre-screen to assess participants susceptibility to motion sickness could be conducted by using the Simulator Sickness Questionnaire^63^ or the Visually Induced Motion Sickness Susceptibility Questionnaire^64^.

The small size of the IVR city and driving on the right-hand side of the road could also have been limitations of our study. The environment with right-hand driving and traffic orientation could have been unfamiliar to our participants from the UK who drive on the left-hand side of the road. However, the participants’ sample was composed by individuals from different countries, as many students and staff were not from UK. Furthermore, the city had no traffic or other vehicles moving and there were no restrictions to participants when driving. No participant showed or reported difficulty in driving during the task or reported finding the right-hand driving unfamiliar and difficult to navigate. Also, all participants underwent a short practice with four trials (one for each GPS condition) to familiarise with the task and IVR environment thus making sure there was a minimum level of familiarity with the right-hand driving for everyone. Finally, all participants took part in all the GPS conditions and thus the differences we found between GPS conditions cannot be accounted for by differences in familiarity with driving orientation among participants. Despite some differences between the GPS instructions used in the present study and those usually provided by real GPS systems, no participant reported difficulty in following the directions, as confirmed by the high level of precision found in the “route deviation” (overall route deviation from the correct/learned encoding route) and non-significant effect of the different GPS systems on this measure. Furthermore, participants reported that they formed a spatial map of the routes to then use during the testing phase. The size of the IVR city was also limited to the assets available to us when the study was developed and carried out and was similar to previous studies that used driving in a virtual city as a way to study memory^52^. However different city generation tools that have been recently released could create a larger city for future studies (e.g., Gaisbauer et al.^65^). A further limitation concerns the unrealistic steering wheel used in the study which was part of the IVR environment. The fact that participants had to control the car without a physical steering wheel and using their hands as acceleration and brake could have increased their cognitive load, thus affecting their responses. Future studies could use a more realistic steering ratio and use a steering wheel to simulate the navigation and increase the ecological validity of the study thus matching more closely the cognitive load in simulated and real driving situations.

Finally, whether our findings transfer to real life is unclear, thus future studies could test whether this multisensory benefit is present during real life navigation with different types of GPS systems. Specifically, the level of redundancy and congruency of audio–visual GPS systems could be examined to assess whether inducing a higher level of multisensory integration in the user could further enhance users’ performance and creation of a cognitive map. In our study we used two common types of audio and visual GPS (i.e., a voice guiding the driver and an arrow moving above a simplified map) which are different types of information that do not match in many ways (e.g., the information may arrive to the user at different times and may have different durations), thus limiting the effect of multisensory integration. One way to increase the match between the type of information provided by the two signals could be to add to the visual GPS system a dynamic changing sound (similarly to what is employed in gaming when cars’ sounds are added in response to the driver behaviour) representing acceleration, deceleration, turns to left and right, which occurs in synch with changes to the arrow movements. Whether such integrated multisensory GPS system would further enhance navigation performance compared to less congruent audio–visual GPS aids and whether this benefit is dependent on drivers’ and users’ spatial orientation preferences and abilities should be examined. The future studies we suggested would allow the creation of integrated multisensory navigation systems that exploit the ability of our brain to combine different sensory cues while taking into account individual differences and preferences in spatial representation.

Finally, while during the visual GPS condition participants could have ignored the visual information from the GPS and relied more on the visual information from the road, during the audio GPS condition participants may have been able to use the GPS information while focusing on the visual information from the road. This is plausibly what occurs when driving in real life using either visual and auditory GPS systems and thus we do not see this as a limitation but rather as an ecological difference between the two types of GPS that could be further examined in future.

## Conclusion

GPS devices are an integral part of our life, they reduce the amount of time in reaching our destinations (e.g., by avoiding traffic jams) and help us to navigate in unfamiliar environments. Our study used an IVR environment to examine whether using a multisensory GPS when driving to a target location in a city could counteract the negative effects of using audio only or visual only GPS during navigation. We specifically examined how different types of GPS affects the formation of a spatial representation or cognitive map and the subsequent navigation performance based on it. We showed that using an audio–visual GPS does improve navigation accuracy and precision when compared to audio or visual GPS alone, however this benefit did not exceed the level of precision and accuracy obtained in the condition with no GPS (although the audio–visual GPS did reduce the time taken to reach the destination compared to when no GPS was available). Hence, using a multisensory GPS can be beneficial to improve navigation performance especially in current times where the majority of people rely on them, and asking them to not use these systems is not feasible.

## Supplementary Information

Supplementary Information 1.

Supplementary Video 1.

## Data Availability

The datasets generated during and/or analysed during the current study are available from the corresponding author on reasonable request.
