# Supplementary material for: Multisensory GPS impact on spatial representation in an immersive virtual reality driving game
Source: Sci Rep. 2022 May 5;12:7401. doi: 10.1038/s41598-022-11124-9 (PMC9072375; doi:10.1038/s41598-022-11124-9)
Supplement: Supplementary file 1 — Supplementary Information 1. [file 41598_2022_11124_MOESM1_ESM.docx]

Multisensory GPS impact on spatial representation in an immersive virtual reality driving game

Laura Seminati^1^, Jacob Hadnett-Hunter^1,2^, Richard Joiner^1^, Karin Petrini^1,3^

^1^Department of Psychology, University of Bath, Claverton Down, Bath BA2 7AY, UK

^2^Department of Computer Science, University of Bath, Claverton Down, Bath BA2 7AY, UK

3CAMERA and REVEAL Research Centers

**Corresponding Author:**

Miss Laura Seminati

Department of Psychology

University of Bath

Claverton Down

Bath

BA2 7AY

United Kingdom

Email: ls2261@bath.ac.uk

**Supplemental material and figures**

**Questionnaire**

Participant n°:

Gender: Male Female

Experience with videogame: Yes No

**Questionnaire on Spatial Representation** (Pazzaglia et al., 2000)

1. Do you think you have a good sense of direction?

1 (not at all) 2 3 4 5 (very good)

2. Are you considered by your family or friends to have a good sense of direction?

1 (not at all) 2 3 4 5 (very much)

3. Think about the way you orient yourself in different environments around you. Would you describe yourself as a person: a. who orients him/herself by remembering routes connecting one place to another?

1 (not at all) 2 3 4 5 (very much)

b. who orients him/herself by looking for well-known landmarks?

1 (not at all) 2 3 4 5 (very much)

c. who tries to create a mental map of the environment?

1 (not at all) 2 3 4 5 (very much)

4. Think of an unfamiliar city. Write the name . . . . . . . . .

Now try to classify your representation of the city:

a. survey representation, that is a map-like representation

1 (not at all) 2 3 4 5 (very much)

b. route representation, based on memorising routes

1 (not at all) 2 3 4 5 (very much)

c. landmark-centred representation, based on memorising single salient landmarks (such as monuments, buildings, crossroads, etc.)

1 (not at all) 2 3 4 5 (very much)

5. When you are in a natural, open environment (mountains, seaside, country) do you naturally individuate cardinal points, that is where north, south, east, and west are?

1 (not at all) 2 3 4 5 (very much)

6. When you are in your city do you naturally individuate cardinal points, that is do you find easily where north, south, east, and west are?

1 (not at all) 2 3 4 5 (very much)

7. Someone is describing for you the route to reach an unfamiliar place. Do you prefer:

a. to make an image of the route?

1 (not at all) 2 3 4 5 (very much)

b. to remember the description verbally?

1 (not at all) 2 3 4 5 (very much)

8. In a complex building (store, museum) do you think spontaneously and easily about your direction in relation to the general structure of the building and the external environment?

1 (not at all) 2 3 4 5 (very much)

9. When you are inside a building can you easily visualise what there is outside the building in the direction you are looking?

1 (not at all) 2 3 4 5 (very much)

10. When you are in an open space and you are required to indicate a compass direction (north-south-east-west), do you:

a. point immediately?

b. need to think before pointing?

c. have difficulty?

11. You are in a complex building (many floors, stairs, corridors) and you have to indicate where the entrance is, do you:

a. point immediately?

b. need to think before pointing?

c. have difficulty?

**Pilot studies description**

Two different pilot studies with 3 participants each, aged between 27 and 30 years (*M* = 28.1, *SD* = 1.16), were carried out to optimise the design and the task used in the main study. The encoding and test phase were exactly the same as in the main study except the differences described below and the number of routes which were ten in these pilot studies.

In a first pilot study participants were not informed of the target landmark they would need to drive to during the testing phase, hence during the encoding phase they could not pay specific attention to this landmark. In a second pilot study, in contrast, participants were informed on what the target landmark was, and thus they could pay specific attention to where the target landmark was during the encoding phase.

Participants in the first pilot study struggled to complete the test phase without knowing what the landmark was during the encoding phase, while the participants in the second pilot had no problem to complete the study, hence we decided to inform the participants about the target landmark before the encoding phase as in pilot study 2. During the pilot phase no participant reported feeling motion sickness and despite the high number of routes they were able to complete the task. As a result the only change we applied to the task was a reduction in number of routes to shorten the study duration and lessen the level of fatigue in participants.

**Screen and headset display resolution**


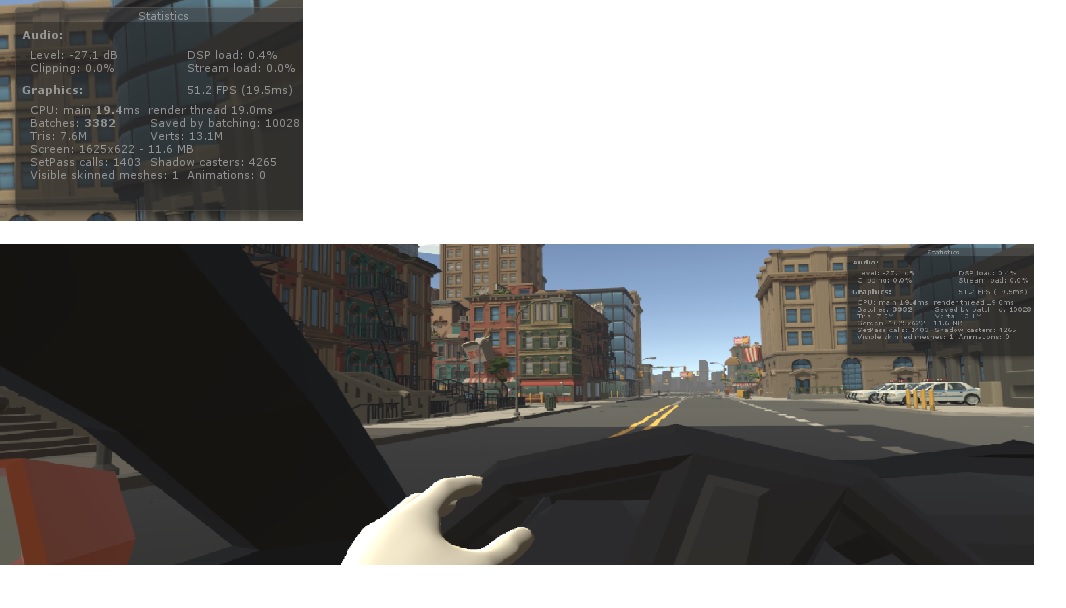


**Figure 1S** Image of the screen from the driver’s viewpoint. On the top left, screen resolution details (1625x622 – 11.6 MB). Note: The Oculus headset display’s resolution was 1080 × 1200 per eye. The experiment was composed by an encoding and a testing phase for each route and GPS conditions. Before each phase task instructions were given to the participants on the screen and the city environment disappeared. This permitted participants to take breaks from holding their hands up, as the virtual car and steering wheel where not visible. This occurred every time they finished a trial and lasted approximately 5-10 sec. Reporting the GPS map in terms of pixels in the VR HMD unfortunately is not very informative. This is because the number of pixels that the GPS map takes up on the VR displays is not fixed. The GPS is a rendered object in 3D space and so its size is dependent of the viewers head position. However, the reader can get an idea of the GPS size by watching the video example for the visual GPS condition.

**Check of normality and sphericity assumptions – Results**

For “end distance” error, fifteen out of twenty (4 GPS x 5 routes) conditions significantly deviated from a normal distribution, *W*(34) ≤ .917, *p* ≤ .013, and Mauchly's test indicated that the assumption of sphericity had been violated for route (*Mauchly's W* (9) = .433, *p* = .002) and GPS*route (*Mauchly's W* (77) = .015, *p* = .001). Similar results were found for “time” and “route deviation”. For “time”, we found that twelve out of twenty (4 GPS x 5 routes) conditions significantly deviated from a normal distribution *W*(34) ≤ .933, *p* ≤ .039, and Mauchly's test indicated that the assumption of sphericity had been violated for route GPS*route (*Mauchly's W* (77) = .009, *p* < .001). For “route deviation”, we found that thirteen out of twenty (4 GPS x 5 routes) conditions significantly deviated from a normal distribution *W*(34) ≤ .931, *p* ≤ .033, and Mauchly's test indicated that the assumption of sphericity had been violated for route (*Mauchly's W* (9) = .305, *p* < .001) and GPS*route (*Mauchly's W* (77) = .011, *p* < .001). Finally, for the “variability” in end distance error (calculated for each participant across the five routes), two out of four (4 GPS) conditions significantly deviated from a normal distribution, *W*(34) ≤ .935, *p* ≤ .044, and Mauchly's test indicated that the assumption of sphericity had been violated for GPS (*Mauchly's W* (5) = .563, *p* = .003).

**Mean rank measures for Friedman’s and Wicolxon’s analyses**

**Table S1. Distance to landmark location**

| GPS conditions | Mean Rank |
| --- | --- |
| noGPS | 2.18 |
| audioGPS | 2.59 |
| visualGPS | 3.06 |
| audiovisualGPS | 2.18 |

**Table S2. Time to landmark**

| GPS condition | Mean Rank |
| --- | --- |
| noGPS | 3.21 |
| audioGPS | 2.26 |
| visualGPS | 2.44 |
| audiovisualGPS | 2.09 |

**Table S3. Variability in distance to landmark location**

|  | | N | Mean Rank | Sum of Ranks |
| --- | --- | --- | --- | --- |
| VISUALGPS - AUDIOVISUALGPS | Negative Ranks | 12^a^ | 12.67 | 152.00 |
|  | Positive Ranks | 22^b^ | 20.14 | 443.00 |
|  | Ties | 0^c^ |  |  |
|  | Total | 34 |  |  |
| AUDIOGPS - AUDIOVISUALGPS | Negative Ranks | 13^d^ | 11.85 | 154.00 |
|  | Positive Ranks | 21^e^ | 21.00 | 441.00 |
|  | Ties | 0^f^ |  |  |
|  | Total | 34 |  |  |

a. VISUALGPS < AUDIOVISUALGPS; b. VISUALGPS > AUDIOVISUALGPS; c. VISUALGPS = AUDIOVISUALGPS; d. AUDIOGPS < AUDIOVISUALGPS; e. AUDIOGPS > AUDIOVISUALGPS; f. AUDIOGPS = AUDIOVISUALGPS
